# Medical students' experiences in providing medical care to older patients: A rich picture study

**DOI:** 10.1111/medu.70181

**Published:** 2026-01-23

**Authors:** Emma J. Draper, Anne de la Croix, Ariadne A. Meiboom, Nynke van Dijk, Rashmi A. Kusurkar, Martin Smalbrugge

**Affiliations:** ^1^ Department of Medicine for Older People Amsterdam UMC location Vrije Universiteit Amsterdam Amsterdam The Netherlands; ^2^ Amsterdam Public Health Aging and Later Life Amsterdam The Netherlands; ^3^ Department of General Practice Amsterdam UMC location University of Amsterdam Amsterdam The Netherlands; ^4^ Research in Education Amsterdam UMC location Vrije Universiteit Amsterdam Amsterdam The Netherlands; ^5^ LEARN! Research Institute for Learning and Education, Faculty of Psychology and Education VU University Amsterdam Amsterdam The Netherlands; ^6^ Amsterdam Public Health Quality of Care Amsterdam The Netherlands; ^7^ Faculty of Health, Sports and Physical Activity, Center of Expertise Urban Vitality Amsterdam University of Applied Sciences Amsterdam The Netherlands

## Abstract

**Introduction:**

With an ageing population, future doctors must be prepared to care for older patients facing complex and often chronic needs. Despite curricular efforts, medical students often report less positive attitudes towards providing this care—shaped not only by knowledge gaps but also by cultural norms and the hidden curriculum. Little is known about how students themselves reflect on their clinical encounters with older patients. This study explores medical students' experiences providing care to older patients, and which aspects they find rewarding or frustrating.

**Methods:**

We conducted a qualitative study based on a constructivist paradigm, using semi‐structured interviews supported by a visual narrative method (rich pictures). Sixteen final‐year medical students who had completed their senior internship were purposively sampled. Participants drew two ‘rich pictures’ representing one positive and one negative clinical experience involving the care of older persons. These drawings were used as prompts for in‐depth interviews. Data were analysed using reflexive thematic analysis.

**Results:**

We identified three themes that captured students' experiences: (1) *feeling connected*, (2) *witnessing humane and compassionate care*, and (3) *making a difference*. Rewarding experiences involved human connection, dignity and presence—particularly in end‐of‐life care or when guided by compassionate role models—leading to a sense of fulfilment. Frustrating experiences arose from poor communication, systemic barriers and unclear goals of care, leaving students feeling powerless, isolated and emotionally burdened.

**Conclusion:**

Students experienced care for older patients as emotionally rich and qualitatively distinct from other clinical work. This practice demands patience, presence, and the ability to navigate complexity beyond mere clinical competence. Medical education should support students in valuing care beyond cure—through fostering reflective practice, peer support, and engaged supervision—helping them reframe what it means to make a difference for older patients and their families in complex, chronic and end‐of‐life care.

## Abstract 2

“Things that aren't described in the textbooks” Medical students' drawings and reflections reveal how care for older patients is shaped by human connection, emotional presence, and systemic barriers. #MedEd

## 1 INTRODUCTION

The global population is ageing, and older adults already account for a substantial proportion of health‐care use.^1^, ^2^ Caring for older patients is therefore a current and increasing reality across most medical specialties. Given their specific health challenges, such as multimorbidity, polypharmacy and complex functional needs, preparing medical students to provide care to this population has become an urgent priority in medical education.

Despite dedicated curricular efforts, research consistently shows that medical students often hold less positive attitudes towards older patients compared to younger ones. For instance, a 2‐week geriatrics clerkship among fourth year US medical students was highly rated but failed to increase their interest in a career in geriatrics.^3^ Such attitudes—viewing older patients as less interesting, less medically challenging or less rewarding to treat—have important consequences. They are associated with reduced empathy, avoidance behaviours and lower interest in geriatric medicine as a career.^4^, ^5^, ^6^

A sociocultural understanding helps explain why such negative attitudes persist. Students' views are shaped not just from a lack of knowledge or skills, but from broader cultural narratives that associate ageing with decline, dependency and societal burden.^7^, ^8^ Personal beliefs, prior contact with older adults and exposure to societal ageism—stereotyping, prejudice and discrimination based on age—affect students' perceptions even before entering medical training.^9^, ^10^, ^11^

Within medical education, these views are further shaped by both the formal and the hidden curriculum: implicit values, behaviours and norms students observe during training.^12^, ^13^, ^14^ When doctors refer to older patients as frustrating or less rewarding to treat, students may internalize the message that caring for older patients is of lower value. Conversely, structured educational interventions, such as geriatric medicine modules, interprofessional learning or longitudinal contact with older adults, can improve competence and attitudes.^15^, ^16^, ^17^ Relationship‐based interventions, where students develop ongoing contact with a community‐dwelling older adult, appear particularly effective in fostering empathy and reducing ageist beliefs.^18^, ^19^

While the influence of the formal and hidden curriculum has been studied, less is known about students' own reflections on their clinical experiences with older patients. Clinical placements – where students encounter both formal teaching and the hidden curriculum—are powerful learning environments in which attitudes are formed and reinforced through observation, participation and reflection.^20^, ^21^, ^22^ Understanding how students interpret these experiences—which aspects they perceive as rewarding or frustrating—could offer valuable insights for educational strategies aiming to improve competence, reduce ageist biases and strengthen professional commitment to caring for older patients.

Therefore, this study aimed to answer the following question: What are medical students' key experiences in providing medical care to older patients, and which elements do they perceive as frustrating or rewarding?

## 2 METHODS

### 2.1 Study design and participants

We conducted an exploratory qualitative study grounded in a constructivist paradigm, which recognizes that knowledge and meaning are co‐constructed through social, cultural and individual experience.^23^ This perspective aligns with our aim to understand how medical students shape their understanding and attitudes toward caring for older patients through their interactions, observations and reflections within clinical learning environments.

The study focussed on 6th‐year medical students who completed their senior internship, the final stage of the Dutch 6‐year medical curriculum (3‐year bachelor's programme followed by a 3‐year clinically oriented master's). During their final internship, students are given more clinical responsibility within a specialty of their choice. Eligible students from the Faculty of Medicine Vrije Universiteit (VU), or University of Amsterdam (UvA), were approached by e‐mail or in person during their final internship. Participants were also asked to recommend other potential participants (i.e. snowball sampling).^24^

To ensure a rich diversity of perspectives, we used purposive sampling to select students varying in gender, age and specialism of their final internship. After six students who had selected geriatrics or elderly care medicine for their final internship were included, subsequent students were only included if they had selected another specialism for their final internship. This was performed to avoid overrepresenting of students with a pre‐existing strong interest in caring for older patients and to minimize response bias.

### 2.2 Data collection

We combined rich pictures—a visual method to explore complexity—with semi‐structured interviews to explore students' experiences in depth. During a rich picture drawing session, the participants are invited to depict a situation and draw their perception of it, with all its interacting components, including things, ideas, people, feelings, beliefs and conflict.^25^ This visual approach encourages the expression of emotions and discussion of complex situations, offering richer insights into students' perspectives.

Each participant was invited to draw two situations from their clinical internships involving the care of older patients: one experienced as positive and one experienced as negative. Drawing sessions lasted approximately 40 minutes and were conducted individually in a quiet room. Immediately afterward, a semi‐structured interview (approximately 60 minutes) used the drawings as prompts to elicit deeper reflection and narrative engagement. Interview questions explored the meaning of the visual representations, such as ‘Could you please explain what is in the picture?’ ‘What did you think/feel/do in this situation?’ ‘What makes it positive or negative for you?’ ‘How did this situation influence you?’

Interviews were conducted by EJD, who had no teaching or assessment role in relation to the participants. Most interviews took place in person at students' own university, so they would feel at ease in a familiar environment; one interview was conducted online using Microsoft Teams because of COVID‐19 restrictions. All interviews were audio‐recorded, transcribed verbatim and pseudonymised. Data collection would be concluded once data sufficiency was achieved, meaning when two subsequent interviews did not yield new insights into the research topics.^26^

### 2.3 Data analysis

Data were analysed using an iterative, reflexive thematic approach.^27^, ^28^ Drawings were used as a prompt for the students to stimulate reflection and were considered contextually but not analysed as a separate dataset. We chose this approach because our focus was on students' interpretations and meaning‐making process, rather than on the visual artefacts themselves. A strength of using rich pictures in this way is that they support emotional expression and help students articulate complex experiences. A limitation, however, is that potential insights from systemic visual analysis were not captured.

The first researcher EJD repeatedly read transcripts and listened to audio recordings, generating initial open codes for two interviews using MAXQDA.^29^ The second researcher AC independently reviewed these transcripts, after which both researchers discussed and refined the codes into a preliminary coding framework. Two additional interviews were then reviewed in the same manner, allowing inductive refinement of the coding scheme and preliminary identification of themes related to the research question.

Subsequent transcripts were coded iteratively by the first researcher, with ongoing discussions with the second researcher to refine codes and interpretations. The full research team periodically met to discuss interpretations, review candidate themes and enhance reflexivity. EJD maintained an audit trail of coding decisions and analytic memos, and used investigator triangulation through independent review and team discussions. Together with the research team, EJD and AC developed narrative descriptions that captured the essence of each theme, illustrated with representative participant quotations.

### 2.4 Ethical considerations

Participation was voluntary. Written informed consent was obtained, and participants were reminded of their right to withdraw at any time. Interviews were audio recorded and transcribed verbatim. Data were pseudonymised and only EJD holding identifiable information. As appreciation, the participants received a small gift (value of 15 euros). The Netherlands Association for Medical Education granted ethical approval (NVMO, file 2020.7.7).

### 2.5 Reflexivity

Our research team members having different perspectives contributed to the enrichment of this study. EJD, AC, AAM, ND, RAK, and MS are researchers in medical education; AAM and MS are trained as medical specialists in elderly care medicine, and EJD, ND, and RAK are trained as medical doctors. EJD, AC, AAM, ND, and MS are medical educators. These varied backgrounds shaped how we engaged with the data, influencing what we noticed, questioned and prioritized.

The team shared a commitment to improving how students learn to care for older patients, which sharpened our awareness of ageism and learning culture. As a junior doctor, EJDs professional proximity to participants likely fostered trust and openness during the interviews. The first researcher's clinical background and the second researcher's non‐clinical perspective enabled mutual questioning of assumptions and potential blind spots. Reflexivity was embedded throughout the whole research process, with regular team discussion supporting critical examination of assumptions and providing analytic rigour.

## 3 RESULTS

We interviewed 16 final‐year medical students, hereafter referred to as ‘students’, from two medical schools (15 from VU and one from UvA) in Amsterdam, between May 2021 and March 2023. Students' background characteristics are summarized in Table 1.

**TABLE 1:** Students' background characteristics.

Age	Range of 23–30 years old
Sex	Women 13 (81%)
	Men 3 (19%)
Specialism final internship	Geriatrics 4 (25%)
	Gynaecology 2 (13%)
	Gastroenterology 2 (13%)
	Elderly care medicine 2 (13%)
	Psychiatry 1 (6%)
	Respiratory medicine 1 (6%)
	Oncology/haematology 1 (6%)
	Oncology 1 (6%)
	Oral and maxillofacial surgery 1 (6%)
	Internal medicine 1 (6%)

We identified three overarching themes that captured the elements students perceived as either rewarding or frustrating when providing medical care to older patients, namely (1) *feeling connected*, (2) *witnessing humane and compassionate care*, and (3) *making a difference*. Below, we elaborate on each theme, supported by illustrative quotes from the student interviews.

### 3.1 Theme 1: Feeling connected

Students described relationships with older patients and their families as central to their experiences. Feeling connected was central to many rewarding experiences, while difficulties in forming such a bond often led to frustration. Recognizing the person behind the patient was essential in forming a connection, but this was sometimes hampered by barriers. Students interacted frequently with family members, whose involvement was perceived as either supportive or challenging.

#### 3.1.1 Seeing the person behind the patient

Many students found that building a personal connection with patients is one of the most rewarding aspects of their experiences. These connections often developed through informal conversations that focussed on patients' lives, careers or interests—bringing out the *‘person behind the patient’* (P9). Shared interests or other similarities further strengthened their bond:

*‘I don't know, but we really had a kind of connection. [...] We got to know each other a bit. And I think, well, that's something special.’* (P12)

Some students admired how some patients coped with illness, describing them as courageous or resilient. Patients, in turn, showed interest in students, making them feel appreciated and seen. Older patients were generally seen as more open to such relationships than younger ones. This was possibly because of having more time and experiencing more loneliness.

Several students noticed the impact of these personal connections on patients. Simple gestures, such as taking time to engage in conversation, could visibly improve a patient's mood: *‘it really does make a difference to someone’* (P12). Students mentioned that older patients expressed more appreciation than younger ones: *‘if you put energy into that, you really get a lot back’* (P16). Feeling connected with a patient motivated students to go the extra mile, such as personally delivering test results at the end of the day.

**FIGURE 1:**
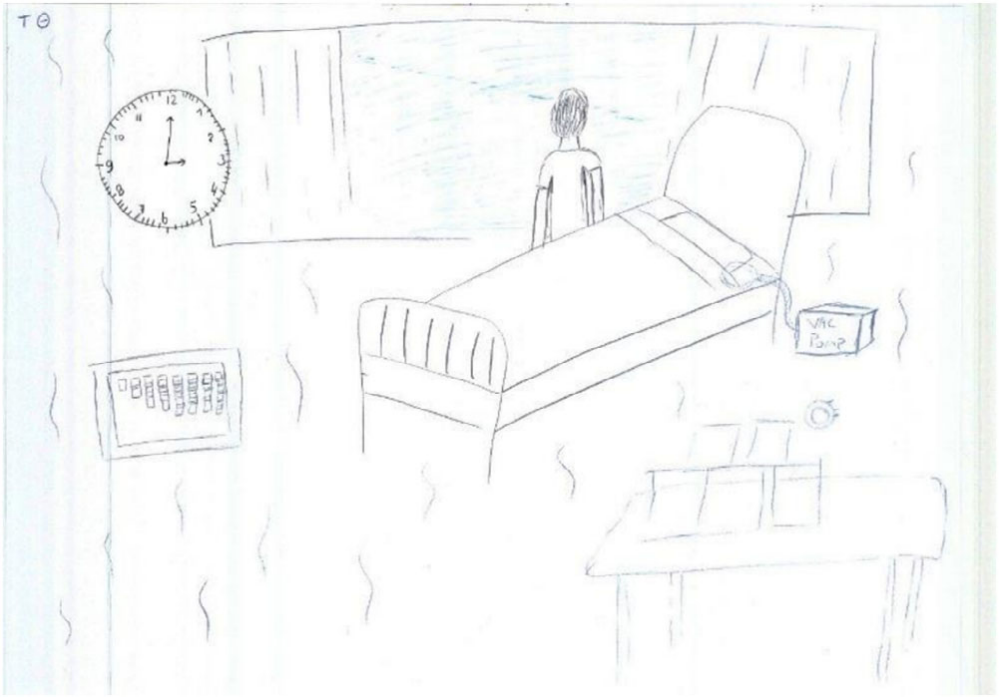
Drawing representing the struggle to establish a personal connection (P7). [Color figure can be viewed at wileyonlinelibrary.com]

In contrast, when connection failed to develop, students often felt frustrated. When a patient's approach to illness or recovery clashed with the student's own views—for example, when patients were perceived as passive or disengaged in their care—students described frustration: *‘I was extremely irritated by this woman’* (P3). Emotional distance could lead to self‐doubt and demotivation. One student, for example, drew a patient with his back turned to her, symbolizing her struggle to connect despite using various conversational techniques (P7; see Figure 1).

#### 3.1.2 Overcoming barriers in communication

While older patients were generally open to conversations, students also encountered barriers in establishing connections, especially because of cognitive impairment such as dementia or stroke‐related aphasia. Overcoming these challenges made the experience even more rewarding.

Some students described efforts to connect with non‐verbal patients through presence, patience and non‐verbal cues:

*‘We got a chair, sat down quietly, and really just took the time and started the conversation, even though we didn't even know if he understood. […] When we looked at photos with him and he laughed […] when you could see in his expression that he understood it or heard you, that was just a good feeling.’* (P4)

Many students found it particularly challenging to feel a connection with patients with dementia. Repetitive conversations and forgetfulness could be frustrating, as they felt they were unable to communicate on the same level:

*‘They would repeat the same things every day and ask the same questions every day. […] So, I got to a point feeling “here we go again” having the same conversation. I just started feeling a bit tired of it.’* (P12)

Some students found ways to connect through humour or involving patients in familiar activities, such as music or games. Others used gentle physical touch to provide reassurance and a sense of presence:

*‘Sometimes just putting your hand on someone's shoulder, or on their hand like, try to stay calm. I get the impression that it helps.’* (P9)

#### 3.1.3 Navigating family dynamics

Interactions with patients' relatives were an important—and often emotionally charged—aspect of students' clinical experiences with older patients.

In several cases, relatives acted as partners in care, particularly when patients were no longer able to make treatment decisions. Involving family members helped to align care with the patient's values, particularly near the end of life, and created a shared sense of purpose:

*‘It is so important that the family supports the treatment plan. […] It really is a process you go through together.’* (P11)

In contrast, difficult interactions arose when there were disagreements regarding treatment goals—particularly when families resisted to accept the care teams' recommendation for a palliative approach. Such situations were experienced as emotionally draining, ethically complex, and could lead to demotivation and avoidance:

*‘At some point, when I saw the family walking through the ward, I'd think: I'll just duck away. Or take another route so I didn't have to walk past their room.’* (P14)

The absence of relatives was also seen as emotionally striking, especially when patients spent their final days alone:

*‘I find his situation sad. That he has no one. No family. […] And that he's really just completely alone.’* (P7)

### 3.2 Theme 2: Witnessing humane and compassionate care

Students found it rewarding to observe older patients receiving humane and compassionate care. Conversely, witnessing inhumane or impersonal treatment—such as rushed consultations or lack of empathy—was frustrating and sometimes altered their overall perception of medical care for older patients. While students felt able to contribute to patients' well‐being, they perceived the broader quality of care as shaped by the culture within the care environment, the health‐care system and the care setting—factors that were beyond their control.

#### 3.2.1 Care culture

Health‐care professionals significantly shaped the prevailing culture within the care environment. Students closely observed how they interacted with older patients, distinguishing between inspiring role models and those they wished to avoid emulating. Time and attention were central to humane care—many students described the importance of visiting patients for ‘*a chat*’.

Some doctors were perceived as treating patients as mere clinical cases. One student described witnessing a rushed doctor treating an older patient as an *‘object’*, who was ignored and neglected (P1). In contrast, a doctor who made time for meaningful contact was seen as a role model: *‘if most GPs did this, or if this happened in care homes, then the care isn't so bad after all’* (P1).

Students especially admired health‐care professionals who went beyond medical responsibilities to bring comfort to patients. For example, a doctor who *‘truly felt for her patients’* made an effort for a patient who missed seeing his pigeons:

*‘At home she made this little thing for on his balcony with bird feed in it, so all those birds would come and he'd still have something to enjoy.’* (P4)

**FIGURE 2:**
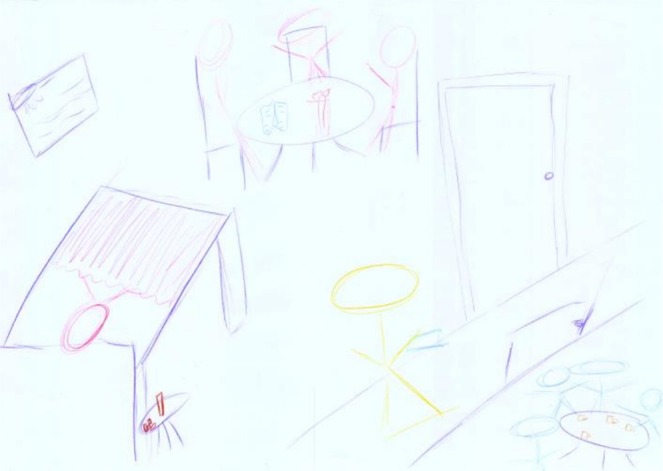
Drawing representing the lack of compassionate presence (P16). [Color figure can be viewed at wileyonlinelibrary.com]

At the end of life, witnessing humane care was even more important. One student described a dying patient receiving adequate medical treatment, but lacking *‘the things that aren't described in the textbooks’* (P16). This student added a hallway with closed doors to her drawing, symbolizing nurses staying in the break room, rather than supporting the dying patient and their family (P16; see Figure 2).

#### 3.2.2 Struggling with the system

Many students encountered systemic constraints that limited their ability to provide the care they felt patients needed. Logistical challenges—such as bed shortages or delays in nursing home placement—demanded time and effort, yet often yielded little result: *‘it's just endless administrative hassle’* (P6). Attempts to arrange appropriate care through various institutions were frequently unsuccessful, leaving students feeling powerless and alone: *‘it kind of remained my problem’* (P6).

Students noted that some medical specialists appeared to have limited interest or experience in caring for older patients, focussing narrowly on their own discipline:

*‘The specialists in [hospital department] they're just absolutely not interested in the elderly. […] They just wanted to deal with the [specific specialty] stuff. […] So they really weren't in the mood for this.’* (P6)

A major issue in providing humane care was time constraints. Older patients often had complex conditions, yet most consultations were brief, and doctors had numerous patients to attend to. One student was initially frustrated by a patient's slowness, but, mid‐sentence, corrected herself—recognizing that the real issue was systemic:

*‘She shuffled into the room very slow with her walker. And straight away it was clear [...] I'm really going to struggle to get this one along in my schedule […] So this was a really unpleasant patient – experience. Actually. Not the patient's fault at all. […] You've got ten minutes to figure something out with someone who doesn't really have the capacity to engage in that way.’* (P10)

Some students described situations in which the health‐care system supported the provision of humane care, such as an in‐depth multidisciplinary meeting or when families helped navigate logistical hurdles.

#### 3.2.3 Setting: atmosphere matters

The physical setting added to how students perceived the quality of care. Nursing homes with personal touches like photographs created a warm atmosphere, maintaining patients' dignity:

*‘Even though they no longer live at home, it's really lovely that they still have their own room.’* (P4)

**FIGURE 3:**
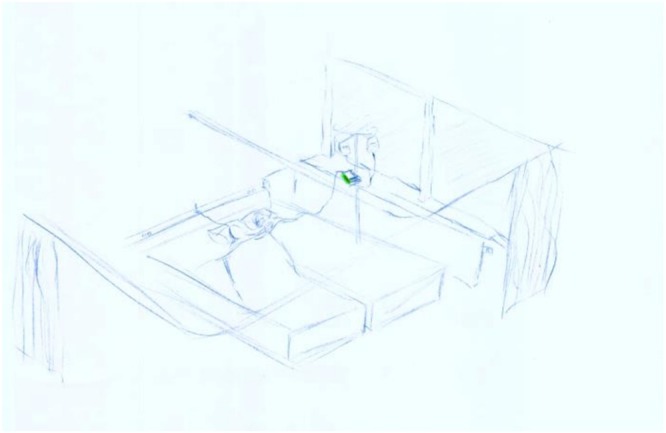
Drawing representing an impersonal and cold care setting (P13). [Color figure can be viewed at wileyonlinelibrary.com]

In contrast, hospitals were often perceived as cold and impersonal. One student drew a dying older patient and grieving spouse sharing their final hours in a large, empty hospital room (P13; see Figure 3):

*‘It was a huge room filled by two small souls.’* (P13)

### 3.3 Theme 3: making a difference

Students' most rewarding experiences involved making a meaningful difference in patients' lives. These experiences rarely involved medical interventions; rather, they were rooted in small, human gestures—being present, listening or responding to personal needs—which seemed to serve as a kind of medicine itself:

*‘If I thought, “I've got half an hour”, I'd go and sit with him. Because you could see, it really did him good […] Even though I couldn't do much for him medically, […] well, I could still do this.’* (P12)

Many students expressed an intrinsic motivation to help others. When caring for older patients, this often meant shifting focus from curing disease to improving quality of life—particularly in the final phases of life. This shift required different skills and attitudes, such as patience, empathy and communication. For some, this brought fulfilment; for others, it led to frustration and discomfort—especially when goals of care were unclear or when they felt unable to contribute to visible improvement.

#### 3.3.1 Shifting the mindset: from cure to care

Caring for older patients prompted students to reconsider what it means to be a doctor. In long‐term care settings, where medical interventions were often limited, some students struggled with abstract goals and felt uncertain about their role:

*‘Honestly, I didn't always know exactly what I was doing in that nursing home. […] I'd thought, ‘what should I actually be doctoring here’?’* (P12)

Some came to value the emphasis on comfort, presence and maintaining dignity. Yet, others perceived the care as lacking urgency and reward:

*‘There wasn't much urgency. […] Even though a woman was lying sick in bed. […] And nothing much happened. That was quite demotivating for me. I was running around like a puppy still wanting to do and see everything. Because it's my first time. But apparently, that's not really needed here.’* (P15)

Navigating between curative and palliative approaches brought challenges, including prognostic uncertainty, family expectations and ethical dilemmas. Students valued doctors who modelled clear, compassionate communication and involved patients and families in shared‐decision making—demonstrating that doctors also make a meaningful impact when curative options are no longer available.

Students who found fulfilment in improving older patients' quality of life accepted the limits of medicine. Instead of viewing the inability to cure as failure, they recognized the value of being present, providing guidance and alleviating discomfort:

*‘With older patients, you can't just ‘fix’ them. There's so much going on, and it's a body in decline. You can't always reverse that. So you need a kind of acceptance that as a doctor you can't fix everything. But you can still mean something to the person in front of you. Even if it's something small.’* (P10)

#### 3.3.2 Finding meaning in end‐of‐life care

Providing care at the end of life was frequently described as deeply rewarding. Students emphasized the importance of presence, compassion, and preserving dignity; when these aspects were absent, it was experienced as distressing. While navigating the transition from curative to palliative care could be complex, the goals of care became clearer in the terminal phase: ensuring comfort and preserving dignity. Death was not perceived as failure, but as a moment to guide with presence and dignity:

*‘When the time comes, I think you just have to let someone go, and try to offer as much comfort as possible.’* (P9)

Being present during these final moments—offering guidance, emotional support or simply listening—was often experienced as a privilege. Witnessing patients surrounded by loved ones was described as deeply rewarding; one student recalled stepping into the room as feeling like a *‘warm embrace’* (P16). Another described how a doctor brought peace to a family by calmly explaining the dying process. One student reflected on the gratitude expressed by family after a patient had passed away:

*‘That I was able to help someone in that phase […] And even though it was a very sad situation [...] I was able to guide them, help them. And really provide a beautiful, yeah, dying moment. Or yes, a lovely end to life, I think.’* (P7)

For many, being present during the dying process was both professionally and personally impactful, becoming aware of the fragility and transience of life. These experiences led to reflection on broader questions about the meaning and quality of life, considering what truly contributes to well‐being:

*‘It's really about the meaning of life. […] That you try to consider as much as possible what contributes to a good life for another person.’* (P9)

Although most experiences were rewarding, a few students described frustrations with end‐of‐life care, such as impersonal settings, difficult communication with families or a lack of compassion from health‐care professionals. One student described the thought of dying in an institutional setting as deeply distressing:

*‘I thought to myself, I would never want to die here. […] Because it's so impersonal. And it's great if you have family, but if you don't you're just lying here dying alone.’* (P16)

## 4 DISCUSSION

Our study adds to the literature a nuanced understanding of how medical students construct meaning around caring for older patients within the sociocultural contexts of clinical training. Students' narratives showed that their attitudes were shaped through interactions with patients, families, other health‐care professionals and other factors from the sociocultural environment. These interactions informed whether they experienced caring for older patients as meaningful, complex or challenging.

Students described caring for older patients as qualitatively different from providing care to other patient populations—requiring not only clinical competence but also patience, presence and the ability to engage with complexity, uncertainty and slower progress. A qualitative study similarly shows that students are particularly drawn to the relational depth and holistic orientation of this care.^30^ In contrast with studies reporting ageist stereotypes or low perceived value, many students found this care meaningful, particularly through providing comfort, dignity or guidance at the end of life.^31^ When students felt equipped and facilitated, this care could be deeply fulfilling; when hindered, they often felt demotivated or frustrated.

Our findings also reinforce evidence that students' attitudes are shaped through social interaction, observation and participation.^32^, ^33^ The relational qualities students valued in role models—such as being caring, empathetic, honest, patient, and involved—mirror what older patients themselves seek in their doctors.^34^ When students were able to practice these behaviours, they experienced it as meaningful and felt acknowledged by patients and families. This underscores how professional values are socially constructed: learners adopt what is modelled, affirmed and made possible within clinical environments.

Frustrating experiences reflected broader systemic and cultural challenges in clinical practice: time pressures, fragmented care and limited training in holistic, person‐centred approaches.^35^, ^36^, ^37^, ^38^ These constraints restricted students' ability to act in ways that aligned with their moral or professional values and affected how they understood their role. Feeling unable to meet patients' needs or witnessing inhumane care undermined their sense of being able to make a difference for a patient. Such feelings of inadequacy or moral discomfort did not stem from personal shortcomings but from organizational and cultural factors that made it difficult to provide the care they valued.

Finally, our findings resonate with Self‐Determination Theory, which posits that intrinsic motivation (in this case for treating older patients) is fostered through experiencing autonomy, competence and relatedness.^39^, ^40^ Rewarding experiences supported these needs, while frustrating ones often undermined them—limiting autonomy (because systemic constraints), diminishing competence (when no solutions were available) and weakening relatedness (through lack of connection with patients, family or teams). Understanding how to support these needs can inform the structuring of clinical experiences and support systems that help students to navigate the emotional and professional demands of providing care to older patients.

### 4.1 Implications for education

This study offers several implications for education.

First, fostering students' sense of connection—through time for dialogue, longitudinal contact or structured reflection—may enhance their learning experiences and resilience. Explicit curricular attention to the relational and humanistic aspects of caring for older patients could help students find meaning in their encounters and strengthen their engagement.

Second, providing space for collective reflection and peer support could help students cope with the emotional demands of providing medical care to older patients. Structured debriefing, group reflection or informal sharing of experiences can reduce feelings of isolation and encourage collaborative problem‐solving—especially in the face of systemic barriers.^41^

Third, engaged supervision of students is important, especially in long‐term care settings where pace and goals of care differ from acute, hospital‐based medicine. Role models who actively discuss palliative care and broader definitions of clinical success can help students appreciate their contributions beyond curative outcomes.

### 4.2 Strengths and limitations

A strength of our study is the richness of student narratives, enhanced by using drawings as prompts to stimulate reflection and expression of emotion. Interviews were conducted by a junior doctor with long‐term care experience—close enough to students' experiences to foster rapport, yet not so senior as to be perceived as intimidating. This helped create a safe and open atmosphere for sharing.

However, our findings may be influenced by response bias, as students with a more positive attitude toward caring for older patients may have been more inclined to participate. The relatively high proportion of students who had chosen geriatrics or elderly care medicine for their final internship supports this possibility. To mitigate this, after six such students were included, we excluded further students from these specialties at the sampling stage. In addition, we asked the participants to describe both rewarding and frustrating experiences.

### 4.3 Future research

Further studies should explore how to best support students in navigating the challenges of providing care to older patients, such as communicating with families, connecting with patients with cognitive impairments and navigating systemic constraints. Future work could also examine how educators can intentionally create conditions that foster more positive learning experiences—such as structured opportunities for meaningful contact, enhanced supervision or guided reflection on complex encounters. A better understanding of this can guide educators in designing curricular interventions that make providing medical care to older patients not only more approachable for students but also more rewarding.

## 5 CONCLUSIONS

Students perceive care for older patients as an emotionally rich and qualitatively distinct medical practice. Students described that human connection, dignity and presence were central in rewarding experiences—whether by developing a personal bond, offering comfort at the end of life or observing compassionate role models. Conversely, frustrating experiences involved poor communication, systemic barriers and unclear goals of care—which inhibited their ability to act according to their moral commitments and led to feelings of powerlessness. These findings highlight that students perceive care for older patients as a complex and meaningful part of medicine that requires more than clinical competence alone. Medical education could support students by fostering reflection, providing engaged supervision and explicitly valuing care that extends beyond cure, thereby enhancing their engagement, competence and professional commitment to caring for older patients.

## AUTHOR CONTRIBUTIONS

**Emma Draper**: Conceptualization, investigation, formal analysis and writing – original draft. **Anne de la Croix**: Formal analysis, writing – review and editing and supervision. **Ariadne Meiboom**: Conceptualization, formal analysis, writing – review and editing and supervision. **Nynke van Dijk**: Conceptualization, formal analysis and writing – review and editing. **Rashmi Kusurkar**: Conceptualization, formal analysis and writing – review and editing. **Martin Smalbrugge**: Conceptualization, formal analysis, writing – review and editing and supervision.

## CONFLICT OF INTEREST STATEMENT

The authors declare no competing interests.

## ETHICS STATEMENT

This study was approved by the NVMO Ethical Review Board under reference number 2020.7.7. Participation was voluntary and informed consent was obtained from all participants prior to data collection. As a token of appreciation, the participants were given a small gift (value of 15 euros).

## Data Availability

The data that support the findings of this study are available on request from the corresponding author. Some data may not be publicly available because of privacy or ethical restrictions
